# ROS, Notch, and Wnt Signaling Pathways: Crosstalk between Three Major Regulators of Cardiovascular Biology

**DOI:** 10.1155/2014/318714

**Published:** 2014-02-04

**Authors:** C. Caliceti, P. Nigro, P. Rizzo, R. Ferrari

**Affiliations:** ^1^Department of Cardiology and Laboratory for Technologies of Advanced Therapies (LTTA Center), University Hospital of Ferrara and Maria Cecilia Hospital, GVM Care & Research, E.S. Health Science Foundation, Cotignola, Italy; ^2^Centro Cardiologico Monzino (IRCCS), Laboratorio di Biologia Vascolare e Medicina Rigenerativa, Milan, Italy

## Abstract

Reactive oxygen species (ROS), traditionally viewed as toxic by-products that cause damage to biomolecules, now are clearly recognized as key modulators in a variety of biological processes and pathological states. The development and regulation of the cardiovascular system require orchestrated activities; Notch and Wnt/**β**-catenin signaling pathways are implicated in many aspects of them, including cardiomyocytes and smooth muscle cells survival, angiogenesis, progenitor cells recruitment and differentiation, arteriovenous specification, vascular cell migration, and cardiac remodelling. Several novel findings regarding the role of ROS in Notch and Wnt/**β**-catenin modulation prompted us to review their emerging function in the cardiovascular system during embryogenesis and postnatally.

## 1. Introduction 

Cardiovascular disease is the number one cause of death worldwide [1]. It has become clear that increases in reactive oxygen species (O_2_^−∙^, H_2_O_2_, and ^∙^OH) represent a common pathogenic mechanism for cardiovascular diseases including atherosclerosis, hypertension, and congestive heart failure [2].

In the last decade the scientific community clarified the importance of low levels of ROS as key signaling molecules in physiological functions such as the regulation of cell signaling, proliferation, and differentiation [3]. Different authors provided scientific evidences regarding a sequential and direct link between Notch and Wnt signaling pathways in tuning endothelial cells (ECs) and cardiomyocytes functions and vascular morphogenesis [4].

The purpose of this review is to describe how ROS regulate Notch and Wnt pathways. Understanding the molecular mechanism regulated by ROS could lead to the development of new therapeutic approaches for cardiovascular diseases.

## 2. Role of ROS in Cardiovascular System

Vascular ROS formation can be stimulated by mechanical stress, environmental factors, platelet-derived growth factor (PDGF), angiotensin II (AngII), and low-density lipoproteins [4–7]. Because many risk factors for coronary artery disease such as hyperlipidemia, hypertension, diabetes, and smoking increase production of ROS, it has been suggested that changes in vessel redox state are a common pathway in the pathogenesis of atherosclerosis [8–10].

A particularly important mechanism for ROS-mediated cardiovascular disease appears to be via stimulation of pro-inflammatory events [11]. These, in turn, cause endothelial cell (ECs) dysfunction that predisposes to atherosclerosis by augmenting thrombosis, inflammation, VSMC growth, and lipid accumulation. The strongest data that link AngII, oxidative stress, and ECs dysfunction are animal studies in which rats were made hypertensive to ~200 mm Hg by infusion of either AngII or norepinephrine. ECs dysfunction was observed only with AngII and correlated with increased superoxide production by arteries [12]. Similar results were obtained from the TREND and HOPE clinical studies which demonstrated that inhibiting AngII restores ECs function and decreases cardiovascular events [13, 14]. More recently, Harrison's lab has shown that AngII stimulates T cell inflammatory responses in a redox-dependent (NAD(P)H oxidases) manner that contributes to hypertension [15].

It has been previously reported that, in response to ROS, VSMC may secrete proteins that participate in autocrine/paracrine growth [16].

ROS are produced at low levels prevalently as byproducts of the mitochondrial electron transport chain and through NAD(P)H oxidase family. They play a physiologically important role in the regulation of various biological responses such as cell migration, proliferation, gene expression, and angiogenesis [17, 18].

NAD(P)H oxidase (Nox) enzymes are membrane-associated enzymatic complexes and structural homologues of phagocytic Nox (gp91phox/Nox2) and consist of both single (Nox1–Nox5) and dual (Duox1 and Duox2) oxidases [19]. ROS are generated by an electron transport through the membrane forming O_2_^−∙^ that can further disproportionate forming hydrogen peroxide (H_2_O_2_) or, in presence of nitric oxide (^∙^NO), creating peroxynitrite (ONOO–).

Nox2 and Nox4 are critical ROS-generating complexes in ECs; they are activated by various stimulants and agonists, for instance, VEGF. Nox1 isoform is more abundant in epithelium, even if it was detected also in ECs and VSMCs [20].

VEGF stimulates ROS production from Nox2 that in turn promotes VEGFR2 autophosphorylation through oxidation/inhibition of phosphatase enzymes. The result is an enhancement in cell proliferation, migration, and angiogenesis [21, 22].

Khatri et al. [23] have shown that vascular Nox2-derived ROS promotes VEGF expression and neovascularization in transgenic mice overexpressing p22phox, a binding partner of Nox. Nox4 is more abundantly expressed compared to Nox2 in ECs. Recently, Datla et al. [24] reported that Nox4 small-interference RNA (siRNA) inhibits VEGF-induced ECs migration and proliferation. Interestingly, Vallet et al. [25] showed that Nox4 expression is upregulated during ischemia-induced angiogenesis of mice. Gene knockout and overexpression studies on Nox4 suggest that Nox4-derived ROS have vascular protective function [26].

Urao and Ushio-Fukai [27] demonstrated that hindlimb ischemia increases Nox2-dependent ROS production in isolated bone marrow-derived mononuclear cells (BM-MNCs) and that postischemic neovascularization and mobilization of BM cells (BMCs) are impaired in Nox2 knockout mice concluding that Nox2-derived ROS regulate progenitor cell expansion and reparative mobilization in response to ischemia.

Furthermore, Nox1-dependent redox signaling pathway modulates the phosphatase PTP-PEST/PTPN12, an important regulator of endothelial cell migration and adhesion [28]. In particular, Nox1-derived ROS were found to promote intestinal mucosa wound repair by inactivating PTEN and PTP-PEST, with consequent activation of focal adhesion kinase (FAK) and paxillin [29].

## 3. Notch Signaling in the Cardiovascular System 

Notch pathway is a highly conserved signaling system that controls cell fate decisions [30]. It is a short range communication system between two adjacent cells based on a ligand-activated receptor. In mammals there are four highly homologous receptors (Notch 1, 2, 3, and 4) and five ligands (Delta-like ligands 1, 3, and 4 and Jagged 1 and 2). Both receptors and ligands are membrane-spanning proteins. Ligand binding induces a conformational change that allows the first proteolytic cut by “A Disintegrin And Metalloprotease,” ADAM (the principal involved are 10 and 17) [31] which removes the extracellular portion of Notch and creates a membrane-tethered intermediate that is a substrate for *γ*-secretase, a cleaving protease complex. *γ*-secretase in turn generates the active form of Notch (Notch intracellular fragment, NIC) which translocates to the nucleus where it binds the transcriptional factor CSL (CBF1, Suppressor of Hairless, Lag-1) also known as recombinant signal binding protein 1 for J*κ* (RBP-J*κ*). Such NIC binding displaces repressor molecules and promotes the recruitment of coactivator molecules. This in turn activates the transcription of specific Notch target genes such as Hes (hairy/enhancer of split), Hey (Hes-related proteins), Nrarp (Notch-regulated andrin repeat protein), cMyc, cyclin D1, and many other genes that control ECs proliferation, differentiation, and apoptosis as well as stem cells maintenance and angiogenesis [32].

Notch receptors 1, 2 as well as Jagged1, Delta-like ligand 1 and 4 (Dll1 and Dll4), are preferentially expressed in ECs and have a key role in developmental and postnatal angiogenesis. During early embryogenesis, Notch induces differentiation of angioblasts to ECs, whereas at later stages it controls specification of ECs into arterial and venous identity [33]. Mice embryos with single Notch1 or double mutations of Notch1 and Notch4 display severe defects in vascular development. In ECs, VEGF-A induces the formation of filopodia, conferring the so-called tip cells phenotype. Tip cells promote a line of angiogenesis while inhibiting branching by adjacent cells (called static or stalk cells) which need to remain angiogenically quiescent. VEGF-A promotes the expression of Dll4 and Notch activation in human ECs, resulting in downregulation of VEGFR2 in tip cells. Thus, this interplay between Notch and VEGF-A regulates angiogenesis with selective sprouting through the activation of the tip cells and suppression of branching through activation of stalk cells. Given its specificity in the regulation of angiogenesis, the endothelial Dll4-mediated Notch signaling has been used as target for intervention in induction of new arteries or inhibition of tumor angiogenesis [34]. VEGFR3, the main receptor for VEGF-C, is also strongly modulated by Notch. Notch inhibition in mouse retina caused an increase in protein levels of VEGFR3 while VEGFR2 levels were unaffected showing that VEGFR2 and VEGFR3 are regulated in a differential manner by Notch [35]. These data have added a new player, VEGFR3, to the model used so far to describe the molecular crosstalk between VEGF and Notch pathways in angiogenesis.

In the context of sprouting angiogenesis, Notch signaling regulates not only the frequency of sprouts but also their ability to anastomose. Retinas of mice heterozygous for a myeloid-specific Notch1 mutation were characterized by long sprouts unable to anastomose and by lack of macrophages at the edge of vascular branch points [36].

Several studies have recognized the central role of Notch-mediated signaling also in cardiac development [37]. In cardiomyocytes, the expression of Notch is not constant over time; it is high in embryonic and proliferating immature cells but disappears when the cells lose the ability to proliferate [38]. Nemir et al. observed that Notch signaling activation inhibits cardiac progenitor cells (CPCs) differentiation into cardiomyocytes [39]. The prolonged activation of Notch in adult cardiomyocytes may have different consequences. Campa et al. observed that in adult cardiomyocytes Notch activation is associated with the block of cell cycle progression and apoptosis, suggesting that a prolonged and uncontrolled activation of Notch can be fatal for these cells [40].

## 4. Wnt/***β***-Catenin Signaling in Cardiovascular System

Notch signaling modulates endothelial homeostasis by crosstalking with other signaling pathways such as receptor tyrosine kinases (i.e., VEGFR2) [41–43] and estrogen receptor [41, 44]. Crosstalk between Notch and Wnt has also been described [41, 45–47]. The Wnt signaling pathway, also called Wnt/*β*-catenin signaling, plays a key role in vascular biology [45, 48]. Mice deficient for Wnt2 displayed vascular abnormalities including defective placental vasculature [49]. Knock-out mice for the Wnt receptor gene, Frizzled5, died in utero due to defects in yolk sac angiogenesis [50]. Defects of the *β*-catenin gene in ECs caused aberrant vascular patterning and increased vascular fragility [51].

The canonical Wnt signaling pathway is driven by *β*-catenin, a scaffold protein, linking the cytoplasmic tail of classical cadherins in the endothelium (vascular endothelial (VE) cadherin and N-cadherin) via *α*-catenin to the actin cytoskeleton. Without Wnt stimulation, cytoplasmic *β*-catenin levels are kept low by a degradation complex, consisting of Axin, Adenomatous polyposis coli (APC), Casein kinase1a (CK1a), and Glycogen synthase kinase 3-*β* (GSK3-*β*). Binding of Wnt to its receptors Frizzled and lipoprotein receptor-related protein (LRP) leads to inhibition of the degradation complex function, enabling *β*-catenin signaling. Wnt allows *β*-catenin to accumulate and translocate to the nucleus where it binds to several transcription factors, for example, T-cell factor (TCF) and LEF-1 [52, 53].

Dishevelled (Dvl) is an essential adaptor protein for Wnt signaling that interacts with several molecules, including Axin, inactivating the *β*-catenin degradation complex [49–52]. Dvl has a dual role: it is an activator of downstream Wnt signaling and an inhibitor of Notch activity. Thus Dv1 is a key regulator of cell-fate decisions in which Wnt and Notch have opposing effects [54].

The noncanonical Wnt pathway can be categorized largely into two classes, the Wnt/Ca^2+^ and Wnt/planar cell polarity (PCP) pathways. The Wnt/Ca^2+^ pathway, mediated by G-protein signaling, stimulates the release of intracellular Ca^2+^ and activation of Ca^2+^-sensitive kinases, such as the protein kinase C and Ca^2+^-calmodulin kinase II. PKC is a family of Ca^2+^-dependent and Ca^2+^-independent isoforms that have different distributions in various blood vessels, and individual members can have different roles in a plethora of biological and pathological events [54–56]. The PCP pathway, also referred to as the Wnt/Jun-N-terminal kinase (JNK) pathway, was originally identified as a pathway affecting cytoskeletal reorganization [57]. This pathway activates small GTPases, including RhoA, Rac, Cdc42, and JNK.

## 5. Crosstalk of Notch and Wnt/***β***-Catenin Signaling Pathways

Notch and Wnt signaling pathways can have opposing effects on cell-fate decisions with each pathway promoting an alternate outcome. For example, in both the skin and mammary gland, Wnt signaling promotes the maintenance of the stem cell fate whereas Notch signaling promotes lineage commitment and differentiation [54, 58–60]. In this case, the inhibition of Notch signaling would help to maintain the stem cell population. The Notch and Wnt pathways also have opposing effects at later steps within a cell lineage. For example, Notch and Wnt signaling influence terminal differentiation within the intestinal epithelium, with Notch activity biasing cells towards the absorptive fate and Wnt signaling favouring secretory cell differentiation [61–64]. Wnt pathway activation promotes neuronal differentiation and inhibits Notch signaling of primary human glioblastoma multiforme- (GBM-) derived cells [65].

Disregulation of Wnt-Notch signaling crosstalk alters early vascular development. Corada et al. [66] found that endothelial-specific early and sustained stabilization of Wnt/*β*-catenin induces activation of the Notch pathway by increasing the transcription of Dll4. This, in turn, prevents a correct endothelial cell differentiation, altering vascular remodeling and arteriovenous specification. Sustained Wnt/*β*-catenin signaling increases Dll4 in tumor vessels and induces Notch signaling that mediates a reduced angiogenic response and normalizes tumor vasculature [67]. Overexpression of Wnt/*β*-catenin signaling leads to alterations of vascular morphology by inhibiting angiogenesis, through stimulation of Dll4/Notch signaling. These effects of *β*-catenin were detectable only during embryonic vasculogenesis and angiogenesis but were lost at late stage of development or postnatally [68].

Yamamizu et al. [69] investigated signal transduction events downstream of the cAMP pathway in embryonic stem cells (ESCs) differentiation and demonstrated that simultaneous activation of Notch and *β*-catenin signaling can constructively reproduce the induction processes of arterial ECs from vascular progenitors expressing VEGFR2 through the formation of an arterial-specific protein complex. In particular these authors reported that RBP-Jk, NICD, and *β*-catenin formed a protein complex that promoted the transcription of genes specifically expressed in arterial but not venous districts in ESCs both *in vitro* and *in vivo*. Moreover, dual induction of NICD and *β*-catenin enhanced promoter activity of Notch target genes *in vitro* (Dll4, Hes1, and EphrinB2) and arterial gene expression during *in vivo* angiogenesis in adult mice.

The finding that a baseline level of Wnt and Notch activity can be detected during vascular development [70–74] and angiogenesis [67] suggests a mechanism in which the level of Wnt and Notch signaling is tightly balanced, that is, through inhibition of Notch/CSL by Dvl [54], and crucial for proper vascular development.

Wnt signaling has been identified as a downstream target of Notch1 that regulates expression of cardiac transcription factors during mouse cardiogenesis and is essential for cardiac development facilitating transcription of target genes involved in cell fate regulation [75, 76]. Kwon et al. [77] recently demonstrated that Notch1 antagonizes Wnt/*β*-catenin signaling by reducing levels of active *β*-catenin in CPCs.

Crosstalk between Notch and Wnt pathways may be partially mediated by specific regulation of GSK3-*β*, a multifunctional kinase that regulates many cellular processes including proliferation, differentiation, and apoptosis [78]. GSK3-*β* is constitutively active in resting cells and is negatively regulated in response to external stimuli by phosphorylation on serine *via* activation of several kinases, including Akt and protein kinase C (PKC) [78]. Endoplasmic reticulum (ER) stress signaling through activation of GSK3*β* is involved in a mechanism of accelerated atherosclerosis in hyperglycemic, hyperhomocysteinemic, and high-fat-fed apolipoprotein E-deficient (apoE^(−/−)^) mouse models; McAlpine et al. showed that atherosclerosis can be attenuated by promoting GSK3*β* phosphorylation [79]. In diabetes mellitus, increased basal GSK3*β* activity contributes to accelerated EPC cellular senescence, effect reversed by small molecule antagonism of GSK3*β* which enhances cell-based therapy following vascular injury [80].

GSK3-*β* directly binds to Notch and inhibits transcriptional activation of different Notch target genes [81–83]. Activated GSK3-*β* reduced NICD degradation by the proteasome, while low GSK3-*β* activity and high Notch signaling correlate with the highly proliferative, undifferentiated nature of EPCs [82]. Phosphorylation of Notch2 by GSK3-*β* is reversed in the presence of Wnt1, resulting in the upregulation of Hes1 [81].

Integrin signaling is linked to Wnt signaling. Rallis et al. showed that, *in vitro*, Integrin-Linked Kinase (ILK) could phosphorylate GSK3-*β* and, in turn, activate Wnt signaling. ILK can also activate Notch signaling. Therefore, the phosphorylation of GSK3-*βvia* ILK directs Wnt and, thereby, Notch signaling activation [84].

On the other hand a recent report [85] showed that GSK3-*β* positively regulates the activity of Notch1 and 3 in VSMCs. Ectopic expression of GSK3-*β* in VSMCs increased NICD levels, promoted CBF-1/RBP-J*κ* transactivation, and enhanced Notch target genes expression. Coincidentally, inhibition of GSK3-*β* activity using a pharmacological inhibitor or reduction in GSK3-*β* levels by siRNA knockdown resulted in attenuation of Notch activity.

## 6. Could ROS Modulate Notch and Wnt/***β***-Catenin Signaling Pathways?

Studies conducted in the last several years have provided clear evidences that both Notch and Wnt/*β*-catenin pathways are regulated at least in part by Nox-derived ROS [86, 87].

Stretch-induced mechanotransduction in VSMCs is known to be regulated by redox signaling initiated by stretch-induced activation of Nox and consequently increased ROS level [88, 89]. In response to stretch, ROS, in particular H_2_O_2_, contribute to the activation of arteriolar myogenic response, contraction, and reorientation, through p38 MAPK signaling activation in VSMCs [90–92]. Zhu et al. [93] showed that cyclic, uniaxial stretch of human VSMCs increased Nox derived-ROS formation and Notch3 activation. Catalase, an antioxidant enzyme that degrades H_2_O_2_, prevented the stretch-induced translocation of Notch3 to the nucleus, increased Hes1 expression, and decreased Notch3 extracellular domain.

In bone marrow-derived mesenchymal stem cells Boopathy et al. [94] observed upregulation of Notch1 and cardiogenic gene expression involving Wnt11 after a myocardial infarction, which induced increased level of H_2_O_2_. These results are in line with other authors: an increase in Wnt11 expression after oxidative stress injury induced cardiomyogenic differentiation of ESCs and mouse bone marrow mononuclear cells [95].

H_2_O_2_ decreases the amount of nuclear *β*-catenin and TCF/LEF-dependent transcription in human embryonic kidney cells. Overexpression of Dvl1 abrogated H_2_O_2_-induced downregulation of *β*-catenin suggesting that H_2_O_2_ negatively modulates Wnt signaling pathway through downregulation of *β*-catenin [96]. A recent study by Funato et al. [87, 97] identified a thioredoxin-related protein, nucleoredoxin (NRX) as a redox sensitive negative regulator of the canonical Wnt signaling through its interaction with Dvl. NRX usually interacts with Dvl but ROS cause dissociation of NRX from Dvl and enable Dvl to activate the downstream Wnt signaling pathway. Furthermore Kajla et al. [98] demonstrated that Wnt treatment of mouse intestinal cells induced ROS production through Nox1 via activation of the Rac1 guanine nucleotide exchange factor Vav2. Nox1-generated ROS oxidize and inactivate NRX, thereby releasing the NRX-dependent suppression of Wnt/*β*-catenin signaling through dissociation of NRX from Dvl. Nox1 siRNA inhibits cell response to Wnt, including stabilization of *β*-catenin, expression of cyclin D1, and c-Myc via the TCF transcription factor.

Msx2, a profibrotic, proosteogenic transcription factor upregulates the expression of multiple Wnt ligands during angiogenesis [99] and enhances aortic canonical Wnt signaling [100]. Msx2-Wnt signaling pathways are induced by tumor necrosis factor *α* (TNF*α*) in arterial myofibroblast via tumor necrosis factor receptor 1 (TNFR1). *In vitro* and *in vivo* gene expression studies established that Nox/mitochondria-derived ROS inhibition reduces Msx2 induction by TNF*α*. Wnt7b as well as *β*-catenin levels were also reduced, suggesting that ROS metabolism contributes to TNF*α* induction of Msx2 and Wnt signaling in myofibroblasts via TNFR1 [101].

APC mutation causes Wnt signaling activation and is commonly found in colorectal cancer [102]. Myant et al. [103] showed that activation of Rac1, a component of Nox protein complex, is required for Wnt-driven intestinal stem cell transformation through ROS production and NF-*κ*B activation. Accordingly, increased ROS might contribute to tumorigenesis by activating specific signaling pathways in different cell types [104], one of which is Wnt signaling.

The cardioprotective effect of ischemic preconditioning (IPC) is abolished by overexpression of secreted frizzled protein 1 (sFRP1), an antagonist of the Wnt/Frizzled pathway, and this effect is related to the ability of sFRP1 to decrease the phosphorylation/inhibition of GSK3-*β* [105]. Cardioprotection involves a link between the mTOR prosurvival pathway and the Wnt pathway *via* ROS that promote activation of Akt and inhibition of GSK3-*β*. The disruption of the Wnt pathway modulates this loop and induces GSK3-*β* activation [106].

Coant et al. [86] reported that direct or indirect redox modulation of the PI3K/Akt and Wnt signaling pathways by Nox1 results in phosphorylation/inhibition of GSK3-*β* and *β*-catenin translocation into the nucleus as well as Notch1 activation. They show that loss of Nox1 results in increased PTEN activity that in turn inhibits Akt signaling pathway, as well as Wnt/*β*-catenin and Notch1 signaling. As previously discussed, GSK3-*β* is an important mediator of Notch-Wnt crosstalk; therefore, it could be a key player by which ROS regulate both pathways.

Taken together these results suggest that redox-dependent regulation of Notch and Wnt/*β*-catenin signaling could provide further insight into their involvement in vascular biology.

## 7. Conclusion and Future Perspectives

ROS have long been deemed as noxious molecules in cardiovascular diseases, including systemic and pulmonary hypertension, atherosclerosis, cardiac hypertrophy, and heart failure. With years of efforts, ROS are becoming increasingly recognized as important modulator for a variety of biological functions and pathophysiological states. Recent evidences suggest an even more significant role of ROS: Notch and Wnt/*β*-catenin signaling modulation. A clear distinction between Notch and Wnt responses is vital for appropriate and robust cell-fate decisions, and ROS modulation of these signaling pathways would provide clues for clinical strategies and drug discovery targeting cardiovascular diseases and cancer.

Above we have discussed how ROS modulation of Notch and Wnt signaling regulates vascular development in different aspects, including stem cells differentiation, angiogenesis, VEGF signaling, endothelial as well as cardiac progenitor cells recruitment, and vascular cell migration. Nonetheless, more details regarding the ROS signaling and pathophysiological functions remain to be elucidated. A deeper insight into the mechanism of how ROS affect normal vascular development, especially CPCs, VSMCs, and ECs differentiation from stem cells, could contribute to a brighter future for regenerative medicine in cardiovascular therapies. Factors that selectively control ROS modulation of Notch and Wnt/*β*-catenin signaling pathways could have therapeutic effects repressing angiogenesis in tumours or favouring it in ischemic tissues.

## Abbreviations

ROS: Reactive oxygen species
Nox: NAD(P)H oxidase
VEGF: Vascular endothelial growth factor
TNF*α*: Tumor necrosis factor *α*
GSK3-*β*: Glycogen synthase kinase 3-*β*
Dll: Delta-like ligand
Hes: Hairy/enhancer of split
Hey: Hes-related proteins
Dvl: Dishevelled
NRX: Nucleoredoxin
HUVECs: Human umbilical vein endothelial cells
EPCs: Endothelial progenitor cells
CPCs: Cardiac progenitor cells
ESCs: Embryonic stem cells
VSMCs: Vascular smooth muscle cells.
